# Impact and applications of cyclic di-GMP second messenger signaling in biotechnology and medicine

**DOI:** 10.1093/sumbio/qvaf016

**Published:** 2025-07-24

**Authors:** Ute Römling

**Affiliations:** Department of Microbiology, Tumor and Cell Biology, Karolinska Institutet, SE-171 77 Stockholm, Sweden

**Keywords:** bacterial biofilms, biodegradation, probiotics, remediation, synthetic biology

## Abstract

3′,3'-cyclic diguanylate (cyclic di-GMP) and alternative cyclic di- and oligonucleotides are ancient highly conserved signaling molecules of bacteria and archaea, which can be present in metazoans up to humans. Their impact in fundamental behavioral modes and physiological and metabolic processes, the modular organization of their signaling cascades and the versatility and flexibility of their components in microbes in combination with their far-reaching effects including stimulation of the innate and adaptive immune response in humans makes these molecules and the respective signaling cascades promising targets in antibiofilm therapy, modulation of multicellularity and tools for treatment strategies and biotechnological applications. This review will thus describe the current state-of-the-art of applications and use as therapeutic targets of cyclic di- and oligonucleotides and the limitations and challenges in the application of those molecules and their use as targets. Future possibilities to successfully exploit those molecules and their signaling cascades equally as potential shortcomings are discussed.

**Sustainability Statement**

Content of this article addresses mainly the Sustainable Development Goal SDG 3 (Good Health and Well-being). In addition, it touches also upon other SDGs, including SDG 6 (Clean Water and Sanitation), SDG 7 (Affordable and Clean Energy), SDG 12 (Responsible Consumption and Production), SDG 1 (No Poverty), and SDG 2 (Zero Hunger). The recognition of global molecular mechanisms of biofilm formation can lead to innovative and effective treatment strategies, novel effective probiotics, and applications addressing SDGs 1, 2, 3 and 7. Equally, the use of microbes and their globally acting signaling molecules and products can aid in the optimization of production processes for natural microbial produced compounds addressing SDGs 7 and 12.

## Introduction

**Figure 1.**
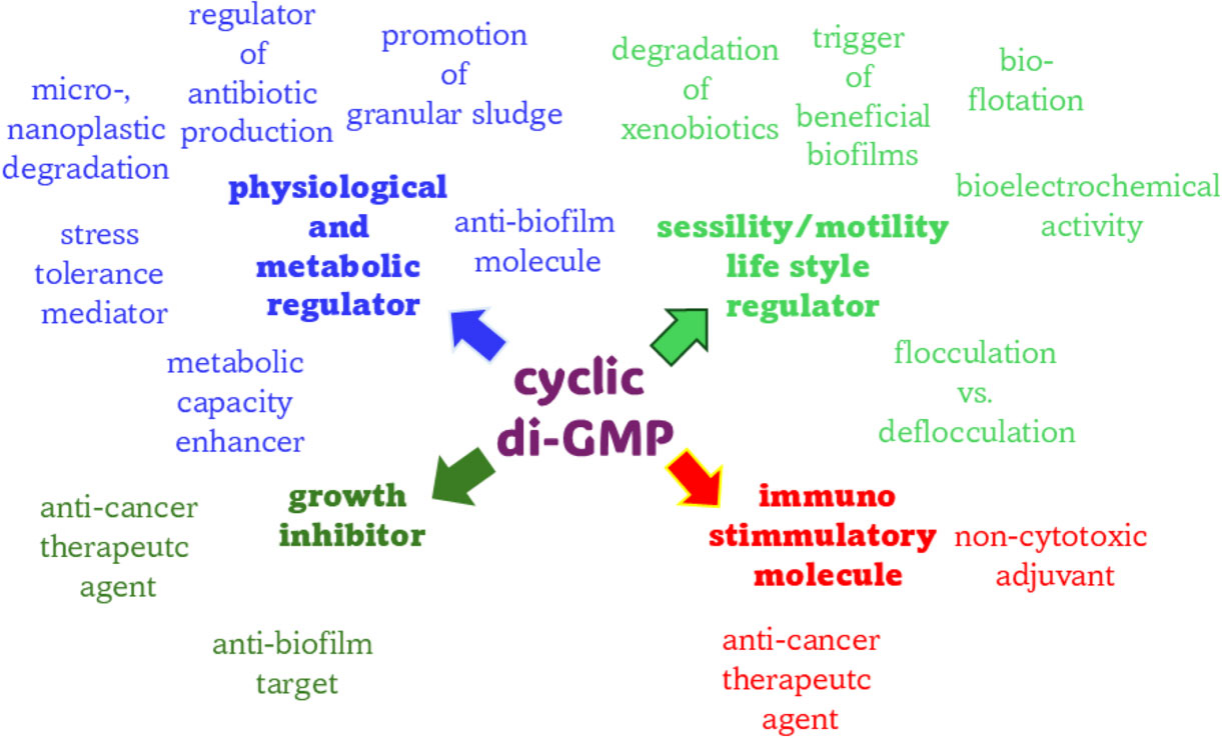
Physiological and metabolic functions of cyclic di-GMP and their exploitation in biotechnological applications and as therapeutic targets.

Although discovered early on (Makman and Sutherland 1965), intracellular second messenger signaling in microbes was for a long time understudied in preference to pheromone-like extracellular quorum sensing signaling pathways (Nealson et al. 1970, Mukherjee and Bassler 2019). However, it is now not only recognized that diverse and complex second messenger signaling pathways exist in bacteria, but also that those pathways can ubiquitously (throughout the phylogenetic tree) direct fundamental physiological and metabolic processes. Recognized to be involved to direct such regulatory functions are, e.g. the cyclic di-nucleotide based second messengers cyclic di-GMP (3′,3′ cyclic diguanylate) and cyclic di-AMP (3′,3′ cyclic diadenylate) (Corrigan and Grundling 2013, Romling et al. 2013). Discovered as the first of its kind as late as in 1987 (Ross et al. 1987), cyclic di-GMP has now been recognized not only as a (nearly) ubiquitous second messenger in Bacteria, but as a molecule that equally ubiquitously directs the single cell life style switch between motility and sessility (used here synonymously with multicellular biofilm formation although not entirely equivalent) (Simm et al. 2004). This regulatory principle is firmly physiologically manifested despite of the conformational flexibility of the molecule, the diversity of cyclic di-GMP binding proteins and their binding sites and the diversity of extracellular biofilm matrix components and their nano-machine like the biosynthesis machines (as well as their associated physiologically, metabolically, regulatory and structurally highly diverse components) in Gram-negative and Gram-positive bacteria. Similarly, cyclic di-AMP has been defined as the molecule to regulate cell wall biogenesis and osmohomeostasis including potassium ion balance (Corrigan et al. 2013) a process that maintains the imbalance of cations as established upon cellular life (Mulkidjanian et al. 2012). Consequently, cyclic di-AMP is found ubiquitously in archaea and bacteria (Braun et al. 2021). As a novel recent twist, cyclic di- and oligonucleotides have been identified as mediator molecules in microbial antiphage defense systems (Whiteley et al. 2019, Kazlauskiene et al. 2017). In this context, cyclic di- and oligonucleotides are mainly produced by the promiscuous cyclic dinucleotide nucleotidyltransferase (CD-NTase) DncV and distinct homologous enzymes [commonly also designated SMODS (Second Messenger Oligonucleotide or Dinucleotide Synthetases)] or Cdns. CD-NTase rarely synthesize cyclic di-GMP, but alternative cyclic di- and oligonucleotides such as cyclic GMP–AMP and cyclic UMP–AMP (3',3'-cyclic uridylate-adenylate). Being signaling proteins in their own right, enzymatic cyclic di-GMP producing GGDEF, DncV, and cyclic di-AMP producing DAC domains belong to three distinct superfamilies, the RNA recognition motif (RRM)-like fold palm domain, DNA-polymerase β-like nucleotidyltransferase and DAC superfamily, respectively (Burroughs et al. 2015, Aravind et al. 2022). Although cyclic di- and oligonucleotides were previously considered to be microbial second messengers with a microbial associated molecular pattern (MAMP) functionality in bacteria and only occasionally introduced in eukaryotes (Chen and Schaap 2012, Cui et al. 2019), this perception has subsequently been challenged by the discovery of 2′,3′-cyclic GMP–AMP as a central innate immune second messenger in metazoans. In this context, cyclic GMP–AMP is produced by cGAS (cyclic GMP–AMP synthase), a homolog of the 3′,3′ cyclic GMP–AMP synthase DncV in bacteria (Zhang et al. 2025a, Davies et al. 2012). Through the cyclic GMP–AMP receptor STING, a broad innate immune response and cell death is triggered (Zhang et al. 2025b). Furthermore, very recent work has shown the human adenylate cyclase 7 to be capable to produce not only cyclic AMP, but also cyclic di-AMP stimulating the NLRP3 inflammasome (Zhang et al. 2025a, Liu et al. 2025). Thus those and other physiological and metabolic functions of cyclic di-nucleotide second messengers, in particular cyclic di-GMP, have opened possibilities to investigate regulatory principles of cyclic di-nucleotide based second messenger signaling in multiple context and for their exploitation in directed ecological, industrial, and agricultural applications and clinical therapies (Fig. 1).

## The cyclic di-GMP signaling system in bacteria

**Figure 2.**
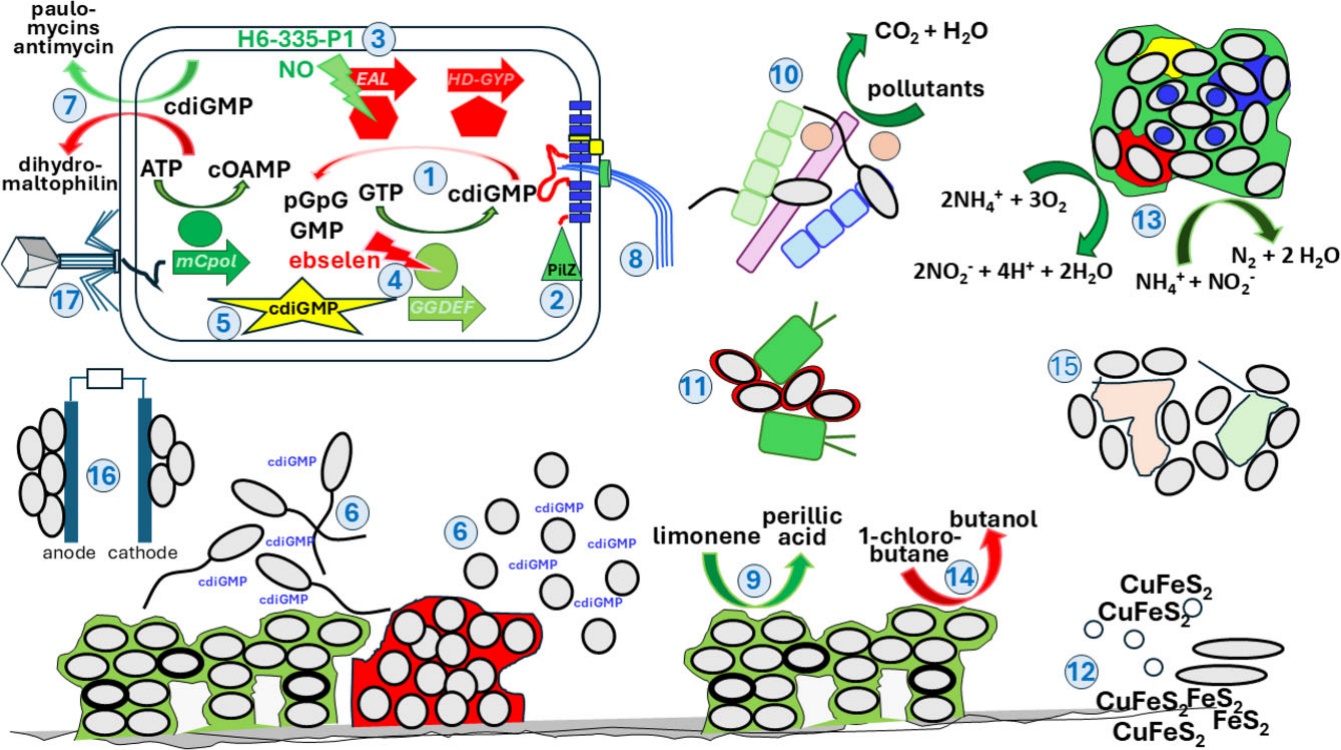
Principles of cyclic di-GMP signaling and different application and interference approaches involving directly and indirectly cyclic di-GMP signaling. (1) GGDEF domain proteins act as diguanylate cyclases and EAL and HD–GYP domain proteins act as phosphodiesterases resulting in pGpG and GMP as products. (2) Receptors/effectors such as the cellulose synthase, which binds cyclic di-GMP via its C-terminal PilZ domain result in the target output biofilm formation, alternatively reduced motility. (3) Antibiofilm molecules enhancing directly (H6-335-P1) or through allosteric interaction upon binding to the signaling domains NosP and H-NOX (volatile nitric oxide NO) the activity of EAL phosphodiesterases are the basis of successful antibiofilm strategies. (4) The seleno-organic compound ebselen and ebselen oxide are antibiofilm molecule broadly targeting I-sites of GGDEF diguanylate cyclases and cyclic di-GMP receptors by covalently binding to the SH-group of cysteine residues as specifically demonstrated for the diguanylate cyclase WspR of *Pseudomonas aeruginosa* (Lieberman et al. 2014, Kim et al. 2021). (5) Cell permeable peptides mimicking a cyclic di-GMP binding site or identified through peptide libraries remove cyclic di-GMP from the signaling pathways as an antibiofilm strategy. (6) Extracellular cyclic di-GMP has been shown to prevent and disperse biofilms of several Gram-positive and Gram-negative organisms and, as also being immunostimulatory, is potentially the basis for antibiofilm strategies. (7) Cyclic di-GMP levels modulate the production of antimicrobial substances such as positively paulomycins and antimycins in *Streptomyces* spp. and negatively dihydromaltiphilin [heat stable antifungal factor (HSAF)] in *Lysobacter enzymogenes*. (8) Cyclic di-GMP regulates cellulose production in many bacterial species, however, only *Komagataeibacter* spp. produce sufficient amounts of cellulose to be used in industrial applications such as the dessert "Nata de Coco" or membranes for speakers. (9) Biofilms, promoted by cyclic di-GMP signaling, can effectively biotransform compounds such as citrus fruit-derived limonene by oxidation to perillic acid, an antineoplastic and antimicrobial compound. Both molecules are natural products but with limited availability (Willrodt et al. 2017, da Costa et al. 2025). (10) Flocs and activated sludge, a loosely connected microbial community, are stress tolerant and contribute to the removal of pollutants from wastewater. As cell aggregation and stress tolerance in liquid culture is a form of biofilm formation, it is hypothesized that cyclic di-GMP signaling contributes to the formation of activated sludge communities. Indeed, major phyla composing the activated sludge, *Zoogloea* and *Thauera* spp. genomes code for cyclic di-GMP turnover proteins (NCBI database results after search with BlastP on July 19, 2025). (11) Flocculation of microorganisms can be applied in various industrial processes, e.g. in the harvesting of microalgae. (12) Differential biofilm formation on minerals and elements such as sulfur contributes to the effective separation and transformation of minerals in the process of bioflotation. Cyclic di-GMP promoted biofilm formation can show enhanced expression of c-type cytochromes (Ng et al. 2020) which contributes to effective redox processes on minerals. In this example, *Leptospirillum ferrooxidans* binds selectively to pyrite (FeS_2_) to promote the effective bioflotation of chalcopyrite (CuFeS_2_) (Bleeze et al. 2018). (13). Aerobic and anaerobic granules are stresstolerant microbial communities tightly connected by exopolysacchrides. Those granules can, e.g. perform a highly efficient denitrification process. (14) Bioremediation, the inactivation of pollutants has been shown to be more effective in (cyclic di-GMP enhanced) biofilms. (15) Degradation of micro- and nanoplastic is more effective if the polymer degrading enzymes are delivered by tightly interacting biofilm-forming microbes. (16) Biofilm formation in electrochemical system either on the anode or cathode contribures to the creation of electric energy and the use of electric energy in valorization of compounds. Cyclic di-GMP and cyclic GMP–AMP has been shown to stimulate biofilm formation in electrochemical systems. (17) Upon phage or plasmid intrusion complementary target transcripts are recognized which activates the GGDEF-evolved nucleotide cyclases Cas10/mCpol to produce cyclic oligoadenylates. Those tri- to hexa-oligoadenylate activate an unspecific RNAse to effectively degrade target RNA (Stella and Marraffini 2024). Further information and references, see main text.

The cyclic di-GMP signaling system is a complex predominantly bacterial second messenger signaling system composed of GGDEF domain diguanylate cyclases and EAL and HD–GYP domain phosphodiesterases as turnover enzymes with the number of encoded proteins grossly linearly correlated with genome size within a phylum (Fig. 2; Schirmer and Jenal 2009, Romling et al. 2013). While cyclic di-GMP signaling overall directs the fundamental life style switch between sessility and motility, each of the, often numerous, individual enzymes can be linked to one or more distinct N-terminally located signaling input domains and can possess a distinct functionality, protein–protein interaction pattern, and regulation despite of the same product output cyclic di-GMP. For example, although biofilms are in general intrinsically tolerant to antimicrobial treatment, a specific GGDEF diguanylate cyclase that mediates antimicrobial tolerance but has no effect on biofilm formation has been identified in *P. aeruginosa* (Poudyal and Sauer 2018).

## Cyclic di-GMP signaling as an antibiofilm target

The original "inventors" of multicellular growth have been microbes, as biofilm formation, the multicellular mode of growth of the autonomously self-sufficiently growing microbial cells, is considered to date back to the origin of cellular life (Shapiro 1998, Martin and Russell 2003, Romling 2023b). Even today, the natural mode of growth of most, if not all microbes at some stage of their life cycle is biofilm formation, the paradigm of tissue formation in higher organisms displaying task distribution and stress tolerance (Fig. 2). On the adverse site, biofilm formation of microorganisms is a major virulence factor of chronic infections, which often leads to treatment failure due to the intrinsic tolerance of biofilms to conventional antimicrobial therapies and the innate and adaptive immune system. As cyclic di-GMP signaling (almost) ubiquitously regulates biofilm formation including in major pathogens such as *P. aeruginosa, Escherichia coli*, and *Acinetobacter baumanii* often causing biofilm-related infections, cyclic di-GMP signaling has been identified as a target for antibiofilm therapies (Opoku-Temeng and Sintim 2017, Sintim and Opoku-Temeng 2020, Liu et al. 2022, Park and Sauer 2022). While the structural heterogeneity and functional redundancy of diguanylate cyclases nearly excludes these enzymes as ubiquitous therapeutic targets for small molecule interference strategies (Fig. 2), such a strategy has been successfully applied to tackle biofilm formation in artificial sputum medium (Cai et al. 2025). In any case, activation of the more promiscuous phosphodiesterases emerged as a promising alternative approach (Fig. 2; Andersen et al. 2021, Hultqvist et al. 2024). Notably, targeting of phosphodiesterases does not only allow the prevention of biofilm formation, but also to efficiently disperse already formed biofilms. Nevertheless, the target of the small molecule interference, the BifA phosphodiesterase, does not seem to be present in all *P. aeruginosa* clones such as the pandemic *P. aeruginosa* clone C (unpublished analysis), thus the ubiquitousness of the approach might not be guaranteed. It remains to be shown whether alternative phosphodiesterases are tackled by the molecule. In an alternative global approach, cyclic di-GMP can be directly scavenged by cell-penetrating peptides resembling high affinity binding sites of protein-based cyclic di-GMP receptors with linear cyclic di-GMP binding motifs (Fig. 2; Hee et al. 2020, Rossmann et al. 2020). On the other hand, unbiased screening of peptide libraries led to the development of a high-affinity peptide pentamer binding dimeric cyclic di-GMP (Foletti, Kramer et al. 2018). A fundamentally different successful strategy to remove cyclic di-GMP from its physiological functions *in vivo* and inhibit biofilm formation to induce quadruplex formation of the molecule which is rarely a recognized ligand (Xuan et al. 2021). Such a strategy might even be more effective if two antibiofilm strategies, scavenging of cyclic di-GMP and production of the antibiofilm agent nitric oxide, NO (see below) are combined (Lin et al. 2023)

Similarly, the defense molecules nitric oxide and the mouse antimicrobial peptide CRAMP are innate immune antibiofilm molecule targeting cyclic di-GMP signaling (Barraud et al. 2006, Zhang et al. 2022b). While also the human equivalent of CRAMP, LL-37, is an antibiofilm molecule preventing the polymerization of the extracellular matrix component curli in *E. coli* (Kai-Larsen et al. 2010, Overhage et al. 2008), its effect on cyclic di-GMP concentrations has not been tested. The NO sensing domains NosP and H-NOX are frequently coupled to interfere with cyclic di-GMP signaling in particular to activate phosphodiesterases (Fig. 2; Barraud et al. 2006, Williams and Boon 2019). While the mechanisms of cyclic di-GMP downregulation are known in the case of NO, the antimicrobial peptide CRAMP downregulates cyclic di-GMP concentration by unknown mechanism(s) (Zhang et al. 2022b). Equally microbial compounds such as quorum sensing molecules, nutrients and metabolites such as Krebs cycle intermediates equally as environmental conditions such as oxygen levels and cation levels can lead to biofilm dispersion with downstream modulation of cyclic di-GMP levels (Rumbaugh and Sauer 2020, Park et al. 2023). On the other hand, first line innate immune molecules such as hypochlorous acid trigger upregulation of cyclic di-GMP via the redox-sensitive CZB domain of the di-guanylate cyclase DgcZ in *E. coli* and, potentially in other bacteria harboring CZB domains in diguanylate cyclases and chemoreceptors (Perkins et al. 2021). As bacteria can also harbor the CZB–EAL domain combination, the possibility exist that the same signal can have an opposite effect in other microbes.

The unbiased search for antibiofilm molecules [molecules that preferentially inhibit biofilm formation compared with growth (IC50_Biofilm_ < IC50_Growth_) or, ideally, are not cytotoxic at all] from natural resources such as from plants, fungi, bacteria, and humans is intensively ongoing and the action of several of them has been associated with the subsequent downregulation of cyclic di-GMP signaling (Kim et al. 2018, Pederson et al. 2018, Ruksakiet et al. 2021). Identification of antibiofilm compounds includes also antibiotics and/or their semisynthetic analogs (Zafar et al. 2020). Among synthetic compounds, the organoselenium compound ebselen and ebselen oxide has been demonstrated to target diguanylate cyclases and I-site GGDEF-based cyclic di-GMP receptors in *P. aeruginosa* to prevent alginate-dependent biofilm formation by covalent modification of cysteine residues (Fig. 2; Lieberman et al. 2014, Kim et al. 2021).

## Cyclic di-GMP application as an antibiofilm treatment strategy

Although cyclic di-GMP signaling is mainly an intracellular second messenger, physiological effect have also been demonstrated when applied extracellularly. One of the most perplexing effects has been the downregulation of biofilm formation even of human pathogens such as Gram-positive *Staphylococcus aureus* not known to harbor a dedicated cyclic di-GMP production pathway (Fig. 2; Karaolis et al. 2005, Yang et al. 2023, Uhlig et al. 2025). This biological activity in combination with cyclic di-GMP's potent adjuvant properties, the substantial activation of innate and adaptive immune responses, can effectively eradicate biofilms and protect against (biofilm) infections (Karaolis et al. 2007, Ogunniyi et al. 2008, Hu et al. 2009). A similar effect of extracellular cyclic di-GMP has also been shown for the Gram-negative gastrointestinal pathogen *Campylobacter jejuni* suggesting the presence of cyclic di-GMP receptors on the surface or the cytoplasmic membrane of the organisms (Elgamoudi et al. 2022).

## Cyclic di-GMP signaling in the regulation of beneficial biofilms

Most biofilms are not detrimental, but beneficial, however the basic mechanisms of cyclic di-GMP signaling in probiotic interactions have been rarely explored. In this context is cyclic di-GMP signaling through its effect on biofilm formation and motility involved in symbiotic interactions between bacterial species and their eukaryotic hosts such as in the case of the bioluminescent bacterium *Vibrio fischeri* with its squid host *Euprymna scolopes* (Isenberg et al. 2024). Noteworthy, recent investigations showed that elevated cyclic di-GMP levels increased colonization of the mouse colon by the probiotic bacterium *Lactiplantibacillus plantarum* by stimulating expression of two mucin-binding proteins (Guo et al. 2025). Equally, a bacterial biofilm consortium that antagonizes *Clostridioides difficile* displays elevated cyclic di-GMP levels (Li et al. 2025). In the plant system, cyclic di-GMP signaling affects biofilm formation and, thus indirectly, biocontrol efficacy of *Bacillus velezensis* PG12, which performs effective biocontrol against apple ring rot disease caused by the fungus *Botryosphaeria dothidea* (Gong et al. 2022) equally as the wheat root colonization capability of *B. velezensis* LQ-3, a biocontrol agent against wheat sharp eyespot. Thus, consequently, although not performed yet, manipulation of the cyclic di-GMP signaling system by natural selection or engineering might lead to more effective probiotics.

## Cyclic di-GMP as a regulator of production of antibiotics

The most successful antimicrobials are natural products produced by microbes or their derivatives. However, their production might be suboptimal or even cryptic under the investigated conditions. Cyclic di-GMP signaling has been found to affect the production of antimicrobials in different directions depending on the organism and the antimicrobial agent opening for the possibility to optimize or even trigger their production by modulation of cyclic di-GMP levels and genetic engineering (Fig. 2; Makitrynskyy et al. 2020, Latta 2022, Fu et al. 2023; Zhang et al. 2025b).

In the predatory Gram-negative bacterium *L. enzymogenes* cyclic di-GMP overall inhibits the synthesis of the heat stable antifungal factor (HSAF) dihydromaltophilin (Liu et al. 2024b, Qian et al. 2020). Receptors of cyclic di-GMP include transcriptional regulators and their interacting components that form a functional complex to modulate the expression of a multitude of genes. Primarily, regulated by a specific pair of diguanylate cyclase/phosphodiesterase cyclic di-GMP suppresses transcription of the HSAF biosynthesis operon by binding to a receptor as part of a functional transcriptional activator complex. Cyclic di-GMP binding leads to the disruption of the protein complex and upregulation of 4-hydroxybenzoic acid, 4-HBA, which consecutively represses transcription further by binding to the now unoccupied transcriptional regulator. On the other hand, however, cyclic di-GMP is required for HSAF secretion through binding to a type 2 secretion system ATPase (Liu et al. 2024b). This concept of dual and even opposite regulation of a specific pathway and/or physiological feature by cyclic di-GMP signaling is also found in the flagellar system reflecting the multiple and even opposite roles that flagella can have on microbial behavior (Römling 2012, Lamprokostopoulou and Römling, 2022).

In *Streptomyces*, the major genus producing antibiotics, cyclic di-GMP is binding as a tetramer to the morphological master regulator BldD that is also directly or indirectly regulating the expression of biosynthetic gene clusters (den Hengst et al. 2010, Tschowri et al. 2014). Thus, among other targets, the production of structurally unrelated antimicrobials such as the phosphoglycolipid moenomycin A, an inhibitor of peptidoglycan transglycosylases, and a cryptic cyclic octapeptide surugamide can be directly or indirectly affected by cyclic di-GMP signaling (Li et al. 2019, Makitrynskyy et al. 2020, Yan et al. 2020).

## Cyclic di-GMP signaling in optimization of new-property-material and biotransformation

Besides antibiotics, bacteria are also a rich source of chemically distinct exopolysaccharides that are exploited in food preparation and processing. Several of these industrially capitalized exopolysaccharides such as alginate and xanthan have been shown to be activated by cyclic di-GMP signaling (Perez-Mendoza and Sanjuan 2016). Cellulose is an exopolysaccharide of multiple bacterial species throughout the phylogenetic tree discovered as early as 1886 (Römling and Galperin 2015, Klemm et al. 2005). Cellulose effectively synthesized by fruit-associated *Komabataeibacter* spp. is an exceptionally pure nanomaterial with high water retention properties commercialized as dressing for chronic wounds and clinically exploited as small-diameter vascular grafts (Fusco et al. 2022, Gürsoy et al. 2025). The ecological and physiological impact of cellulose production for *Komagataeibacter* spp. is manifested genetically as their chromosomes can encode multiple cellulose biosynthesis operons with associated cyclic di-GMP turnover genes whereby different strains produce cellulose with unique properties (Ryngajllo et al. 2019). The modular organization of cellulose biosynthesis genes opens up for tailor-made engineering of cellulose macromolecules with unique properties (Goosens et al. 2021, Bimmer et al. 2022, Bimmer et al. 2023). Thereby cyclic di-GMP that post-translationally activates cellulose biosynthesis via the C-terminal PilZ domain of the BcsA cellulose synthases boost high cellulose production levels (Fig. 2; Tal et al. 1998).

While antibiotics and exopolysaccharides are bioactive compounds of therapeutic value intrinsically produced mainly by bacteria, microbes can also carry out distinct chemical reactions upon the provision of precursor compounds in a process called biotransformation (Fig. 2; da Costa et al. 2025). Immobilized microbes (in biofilms) can more efficiently transform substrates and are readily separated from the desired product. Although not formally shown, it is suggested that manipulation of the cyclic di-GMP signaling pathway will aid in the immobilization of the microbes and potentially also improve the biotransformation of the substrates.

## Potential role of cyclic di-GMP signaling in flocculation—for flocculants—in flotation

Microbial flocculation, the formation of multicellular single or multispecies loosely connected ggregates, is a variant mode of biofilm formation with ecological and industrial impact (Schleheck et al. 2009, Srivastava et al. 2018). Flocculating microbes, may they consist of a single isolate or self-associated consortia, display biofilm-like features including the promotion of stress tolerance. Their bio-flocculation capacity and associated unique metabolic and physiological properties are used in various industrial applications such as wastewater and industrial sludge treatment, co-culturing and harvesting of microalgae, industrial ethanol production, carrier material, bioleaching of metals, and facilitate the removal of microorganisms from their end products (Fig. 2; Arcuri 1982, Yu et al. 2023, Ayata et al. 2024). Aerobic granules, with granules described below, microbial entities with similar, but enhanced physiological and metabolic properties compared to activated sludge (flocs), can serve similar function such as being a carrier material for hydrogen producing *Caldicellulosiruptor* spp. (Fig. 2; Wan et al. 2014, Pawar et al. 2015). As another example, the alpha-proteobacterium *Zymomonas mobilis* has been traditionally used for >1500 years for the production of alcoholic beverages. In order to maximize productivity in industrial ethanol production, flocculating ethanol tolerant variants of *Z. mobilis* have been selected (Arcuri 1982, Lee et al. 1982, Skotnicki et al. 1982). Notably, genomic and functional analyses of strain *Z. mobilis* ZM4 and its flocculating variant ZM401 revealed mutations addressing the production the biofilm extracellular matrix component, the exopolysaccharide cellulose, and cyclic di-GMP signaling to trigger the flocculant phenotype (Cao et al. 2022). On the other hand, cell aggregates and biofilm formation cause membrane biofouling thus dispersal of flocs might be desirable in other industrial processes (Chen et al. 2022). Such dissolution of biofilms can be promoted by cyclic di-GMP specific phosphodiesterases.

A prerequisite for flocculation and a to-be-exploited byproduct of microbial flocculation is the production of flocculants, secreted microbial extracellular matrix components of different chemical nature (Rebah et al. 2018, Liu et al. 2021). These industrially applicable microbial flocculants, intended to replace inorganic and organic polymeric flocculants, with their application mainly hampered by the high production costs, are produced by diverse microbes, including bacterial strains, in order to act in processes as diverse as a suitable matrix for nanoparticle biosynthesis to heavy metal harvesting and removal and recovery of sulfur compounds.

In conclusion, in general, flocculating and flocculant producing strain variants of bacterial species might possess a specific constellation of cyclic di-GMP turnover proteins, which enables mono- or multispecies cell aggregation in liquid culture, flocculation, and flocculant production, under the investigated conditions. Considering this hypothesis, cyclic di-GMP signaling is not only a subject to be investigated in those isolates, but also a target to engineer flocculating and stress tolerant strains equally as strains overproducing flocculants for different industrial applications.

Bioflotation, the harvest of minerals by microorganisms and their products by either microbe-mineral and microbial product-mineral or microbial metabolism-mineral interaction is another biofilm-associated bioprocess (Fig. 2; Kinnunen et al. 2020, Ayata et al. 2024) where manipulation of the cyclic di-GMP signaling system in applied microbes can presumably enhance performance. Cyclic di-GMP signaling has been shown to promote biofilm formation on minerals and sulfur promoting biogeochemical processes such as sulfur oxidation (Ruiz et al. 2012, Castro et al. 2015, Diaz et al. 2018). In conclusion, as life developed in close interaction with inorganic surfaces (Römling 2023a, Römling et al. 2023, Martin et al. 2008, Martin and Russell 2003, Mulkidjanian et al. 2012), these industrial processes mimic the ancient and still present tight microbial interactions and chemical processes with abiotic minerals.

## Cyclic di-GMP signaling in wastewater treatment

Efficient wastewater treatment aims at the removal of pollutants, nitrogen, phosphorous, heavy metals, and other contaminants that impair water quality. Thereby, biofilms and activated sludge, spontaneously derived consortia of sessile and floating microbial communities, respectively, are conventionally used for this objective. Due to their purpose, exopolysaccharide producing and nitrogen and phosphate removing bacterial genera such as *Zoogloea, Thauera, Dokdonella*, and *Nitrospira* spp. dominate. To remove nitrogen in the form of ammonia, combining ammonia oxidizing bacterial communities with anaerobic ammonium oxidating (anammox) communities mainly consisting of microbes belonging to the Planctomycetota phylum creates a substantially resource and energy saving process for efficient denitrification (Fig. 2). In this and other context, granular sludge, such as aerobic or anaerobic granular sludge, tightly aggregating microbial communities characterized by the excessive production of extracellular polymeric substances (EPS) demonstrate superior performance in (experimental) wastewater treatment including high toxicity tolerance and settleability (Fig. 2; Hulshoff Pol et al. 2004, Nancharaiah and Kiran 2018). Formation of granular sludge, its improved formation and its stability against challenges has been consistently correlated with alternating concentrations of cyclic di-GMP and EPS production concomitantly also with shifts in the microbiota (Wan et al. 2013). For example, stabilization of different aerobic granular sludges concomitantly with elevation of cyclic di-GMP and EPS levels was achieved by adding additional components as diverse as nitrate, salicylic acid, spermidine, high NaCl concentration, a quorum sensing diffusible signaling factor inhibitor, nitrogen dopped graphene, zerovalent iron, and a combination of *Aspergillus tubinginsis* mycelium pellets combined with Fe_3_O_4_ nanoparticles (Zhang et al. 2022a, Wan et al. 2013, Chen et al. 2020, Li et al. 2020, Wu et al. 2023, Lin et al. 2024, Tang et al. 2025). Exposure to Mn^2+^ ions and heavy metal contamination was subsequently found to destabilize aerobic granular sludge including downregulation of cyclic di-GMP and EPS although initially efficient in removal of the divalent cation (Liu et al. 2024a).

Although signaling events and causal mechanisms leading to the transition from activated to granular sludge remain to be unraveled, members of the microbial communities forming the granular sludge have been demonstrated to encode functional cyclic di-GMP turnover proteins (Zhang et al. 2022a, Guo et al. 2017). Understanding regulatory processes of sludge and granule formation might aid in the rational design of effective strategies to direct the formation of tailor-suited effective and diverse microbial communities ideally by a combination of nutritional triggers and signaling thus avoiding the construction and release of diverse genetically engineered microbial communities, which might even prove unstable in the natural ecological settings.

## Cyclic di-GMP as a regulator of xenobiotic degradation

Microbial biofilms are tolerant against stress compounds including xenobiotics. These features together with a higher metabolic capacity to degrade xenobiotics make biofilms to ideal candidates for bioremediation processes (Fig. 2; Bhatt et al. 2023, Sarkar and Bhattacharjee 2025). While biofilm formation of microorganisms involved in bioremediation such as *Pseudomonas* spp. and *Burkholderia* spp. are regulated by cyclic di-GMP signaling, several examples have already demonstrated a performance-improving role of cyclic di-GMP signaling. Engineered xenobiotic-responsive expression systems showed more effective degradation of 1-chlorobutan in biofilm versus planktonic cells (Benedetti, de Lorenzo et al. 2016). As another example, dibenzothiophene (DBT) is refractory to biodesulfurization. Concomitant induction of the DBT desulfurizing gene cluster *dszBACD* and cyclic di-GMP dependent biofilm formation in *Rhodococcus erythropolis* IGTS8, however, led to the effective desulfurization of DBT (Dorado-Morales et al. 2021).

Purification of water and ground water involves sand filters that are bioaugmented with microorganisms for efficient removal of xenobiotics and heavy metals (Ellegaard-Jensen and Gobbi et al. 2020). Manganese (II) ions to be removed from ground water due to cosmetic and taste issues have been shown to be efficiently oxidized by a cyclic di-GMP regulated peroxidase from a *Pseudomonas resinovorans* biofilm (Piazza et al. 2022).

## Cyclic di-GMP and biofilm based strategies to degrade micro- and nanoplastic

With 400 million tons (numbers for 2022) of plastic, a common designation for polyethylene, polypropylene, polyethylene terephthalate (PET), polystyrene, polyvinyl chloride, and other synthetic polymers, produced annually, a substantial fraction of 8 million tons of this material ends up in the ocean alone (Jambeck et al. 2015). Although biodegradable capability of microbes for plastic material has been observed (Emadian et al. 2017), hard to degrade micro- and nanoplastic can persist in great numbers in the environment at least for decades. The high number of particles have substantial ecosystem modulating capability as microbial biofilms consisting of unique community compositions are readily formed on the surface (Stabnikova et al. 2021, He et al. 2022). Such is the microbial community on plastic less diverse, microorganisms colonizing microplastic displayed enhanced virulence equally as natural transformation suggesting the accelerated spread of antimicrobial resistance (Tang et al. 2022, Wang et al. 2023, Nath et al. 2025). Considerable advances have been made in order to identify and characterize degrading enzymes for different types of plastic (Tournier et al. 2023). With plastic degradation enzymes secreted by biofilm-forming microbes on the plastic surface, cyclic di-GMP enhanced biofilm formation leads to a more effective degradation of PET (Howard and McCarthy 2023), but also can cause subsequent dispersal by plastic degradation products (Preuss et al. 2025). This biofilm and cyclic di-GMP based strategy for the close and concentrated delivery of plastic-degrading enzymes for the degradation of environmental plastic can certainly be further modulated and optimized (Fig. 2).

## Cyclic di-nucleotide mediated biofilm formation in bioelectrochemical systems

Microbial fuel cells, biological systems to create electric energy, belong to the bioelectrochemical systems that are based on electroactive biofilm formation of microorganisms (Fig. 2; Greenman et al. 2021). Two of the most abundant organisms and most frequently used model microbes in pure culture in microbial fuel cells are *Geobacter* spp. and *Shewanella* spp. due to their superior capacity to effectively transfer electrons via nanowires, nanotubes, terminal reductases, and extracellular small molecule electron carriers obtained from the oxidation of, e.g. wastewater products, to the colonized anode (Lovley and Holmes 2022) combined with metabolic versatility. Cathodic biofilms, on the other hand, will reduce oxidizing substrates such as oxygen with obtained electrons (Jain 2018). However, the biofilm on the cathode can also be used for bioreduction of compounds such as CO and CO_2_ in bioelectrosynthesis processes.

Cyclic GMP–AMP is a cyclic di-nucleotide that can be synthesized either by a subgroup of GGDEF domains (Hallberg et al. 2019) or by CD-NTase/SMODS a distinct class of promiscuous di- and oligonucleotide cyclases with *Vibrio cholerae* 3′,3′-cyclic GMP–AMP synthesizing DncV as the prototype (Davies et al. 2012, Burroughs et al. 2015). Cyclic GMP–AMP as a second messenger can have distinct, yet overlapping functions compared to cyclic di-GMP mediated by specific receptors (Li et al. 2019, Lowry et al. 2022). Notably both cyclic GMP–AMP and cyclic di-GMP can promote formation of electroactive biofilms, with ectopic regulation of cyclic di-GMP to show a differential effect (Nelson et al. 2015, Ng et al. 2020, Hu et al. 2024, Jiang et al. 2025). Indeed, genetic engineering of *Shewanella oneidensis* to improve colonization and electron transfer capacity including enhancement of biofilm formation by boosting cyclic di-GMP levels led to a significant improvement of electroactivity with an almost 40-fold higher output power (Li et al. 2023).

To extend these findings to practical applications, cyclic di-GMP mediated enhancement of biofilm formation in engineered bacteria is also expected to improve the efficiency of microbial electrolysis cells, which use the oxidation of xenobiotics from wastewater to transfer electrons for the subsequent production of hydrogen or methane and microbial desalination. On the other hand, desalination of water with reverse osmosis including nanofiltration and electrodialysis requires membranes not clogged by colonizing biofilms.

## Cyclic di-nucleotides to restrict phage infection

**Figure 3.**
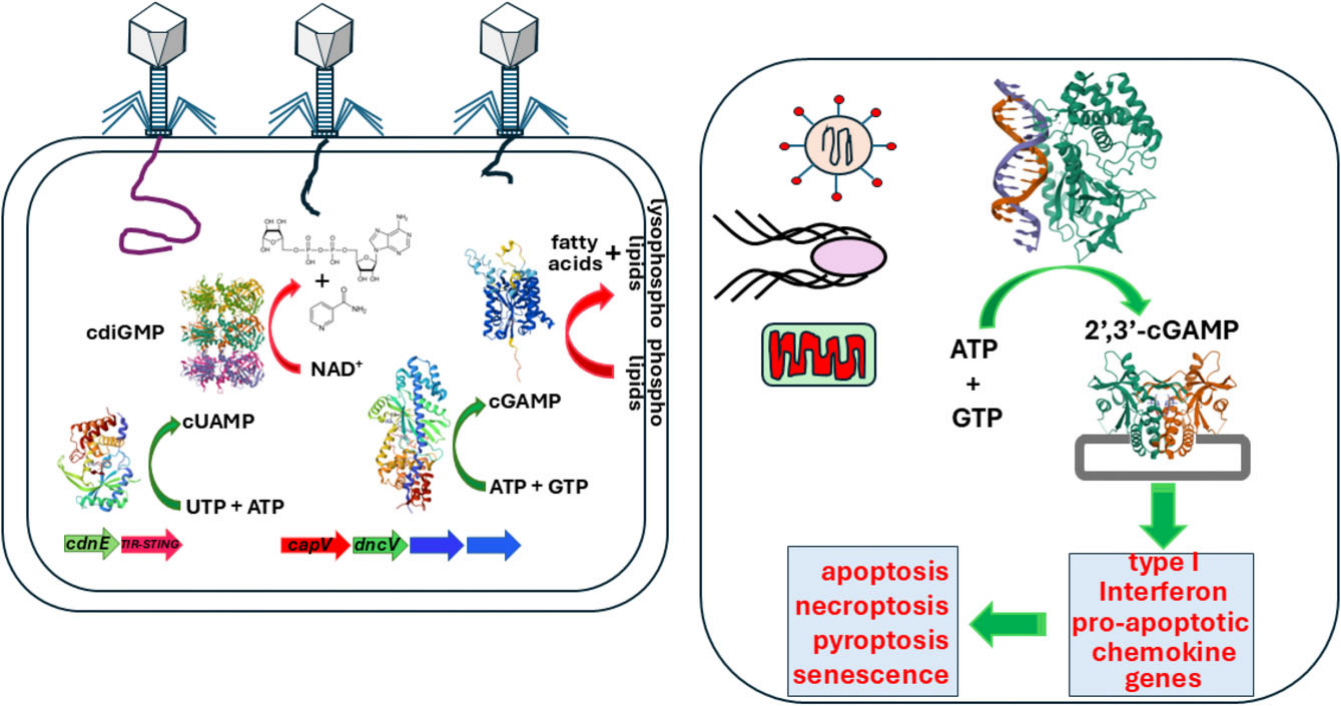
Basic principles of cyclic di-nucleotide signaling in microbes compared to metazoans (humans). Left, basic mechanisms of CBASS signaling in bacteria. Upon phage infection, DncV and DncV-like dinucleotide cyclases (CD-NTases) are activated to synthesize different cyclic di- or oligonucleotides. There exist 10 phylogenetically deeply branching clades of Cdns, CdnA (DncV) to CdnH (Whiteley et al. 2019). While CdnA synthesizes cyclic GMP–AMP, CdnE-like enzymes can synthesize cyclic UMP–AMP, cyclic di-GMP and other cyclic di-nucleotides. Cyclic di- and oligonucleotides subsequently binding to receptors, conventionally effector proteins such as the patatin-like phospholipase CapV or the TIR-STING enzyme provoking cell lysis and energy depletion, respectively. CapV catalyzes the hydrolysis of phospholipids into lysophospholipids and fatty acids, while Tir-STING can bind cyclic di-GMP via the STING domain to provoke hydrolysis of the reduction equivalent NAD^+^ into nicotinamide and adenine diphosphate ribose, another second messenger. Figures of crystal structures of DncV, CdnE, and TIR–STING are taken from the PDB database (4U03, 6E0O, and 7UN8, respectively), while the Alpha-Fold derived structural model of CapV (AF-P0DTE9-F1) was taken from UniProt (UniProt Consortium 2018). Right, major signaling pathway for 2′,3.-cyclic GMP–AMP in metazoans (humans). DNA derived from viruses, bacteria, and intrinsically from mitochondria activate the cyclic GMP–AMP synthase cGAS. Cyclic GMP–AMP subsequently binds to the membrane-bound endoplasmatic reticulum receptor STING to provoke a broad innate immune response with, e.g. the transcriptional activation of type I interferon genes. This transcriptional regulation can lead to apoptosis, necroptosis, pyroptosis, and senescence of cells (Decout et al. 2021). Pictures of crystal structures of cGAS in complex with DNA and STING are taken from PDB [6CT9 and 4KSY, respectively (Zhang et al. 2013, Zhou et al. 2018)]. Further information and references, see main text.

Besides being a major regulator of the life style switch between sessility and motility, cyclic di-GMP, but predominantly alternative cyclic di- and oligonucleotides such as cyclic UMP–AMP and cyclic AMP–AMP–GMP have been identified as second messengers in innate and adaptive phage and plasmid defense systems such as the cyclic oligonucleotide-based antiphage signaling system (CBASS) and CRISPR–Cas system, respectively (Burroughs et al. 2015, Kazlauskiene et al. 2017, Severin et al. 2018, Whiteley et al. 2019, Duncan-Lowey et al. 2021, Duncan-Lowey and Kranzusch 2022). In the CBASS innate immunity phage defense system, synthesized by distinct DncV-like CD-NTase/SMODS belonging to the DNA-polymerase β-like superfamily, cyclic di-, and oligonucleotides subsequently bind to (co-localized) effectors, which causes growth retardation, cell lysis and prevention of phage propagation by distinct mechanisms such as activation of patatin-like phospholipases and enzymatic Toll/interleukin-1 receptor (TIR) domains that hydrolyse the reduction-equivalent nicotinamide adenine dinucleotide NAD^+^ (Fig. 3).

The remarkable near-to-absence of cyclic di-GMP signaling in the Bacteroidota (previously Bacteriodetes) phylum indicates its predominant role in CBASS antiphage defense rather than regulation of the motility/biofilm switch (Morehouse et al. 2020). It has thus been hypothesized that involvement of cyclic di-GMP/cyclic di-nucleotide signaling in antiphage defense systems is mutually exclusive with a physiologically relevant cyclic di-GMP signaling system that directs the motility/sessility physiological responses. Indeed, the expression of some GGDEF diguanylate cyclases and the expression of deregulated protein mutants in certain bacterial strains such as in the *E. coli* B strain BL21 has been shown to be cytotoxic in a cyclic di-GMP-dependent manner (Christen et al. 2006, Liu et al. 2018), with lack of cyclic di-GMP to abolish the motility/sessility cell cycle but not growth (Abel et al. 2013). Cyclic di-AMP, however, is ubiquitously cytotoxic upon overexpression and deletion (Corrigan and Grundling 2013). In other context, cyclic di-GMP signaling and its target output have, however, been shown to promote phage infection by activating the synthesis of their exopolysaccharide receptors (Mutalik et al. 2020, Junkermeier and Hengge 2021, Sellner et al. 2021, Knecht et al. 2022, Walton et al. 2025). Furthermore, cyclic di-GMP has been shown to be an antitoxin (Liao et al. 2024). Last, but not least, type III CRISPR–Cas system Cas10 containing an evolved GGDEF domain (Cas10/mCpol) synthesizes cyclic oligo-adenylates in response to target RNA recognition in order to activate the unspecific ribonuclease Csm6/Csx1 (Fig. 2; Kazlauskiene et al. 2017, Niewoehner et al. 2017). Cumulatively, these findings indicate that interactions between phages and cyclic di- and oligonucleotides signaling pathways are complex, but can be used as regulatory tools to modulate phage innate and adaptive immunity equally as to optimize phage cocktails for phage therapy.

## Cyclic di-GMP as adjuvant

Long before the concept of pathogen associated molecular patterns and interkingdom crosstalk have been ubiquitously accepted, first indications that cyclic di-GMP stimulates the immune response and selectively affects the growth of cancer cells have been reported (Amikam et al. 1995, Steinberger et al. 1999). These observations have been later independently confirmed (Karaolis et al. 2005, Karaolis et al. 2007, Ogunniyi et al. 2008) leading to the recognition of cyclic di-GMP and its analogs as efficient noncytotoxic adjuvants and as compounds for cancer treatment (Ebensen et al. 2007, Chandra et al. 2014, Miyabe et al. 2014).

First reports on the impact of cyclic di-GMP on host cells applied unphysiologically high concentrations of the cyclic di-nucleotide. However, cyclic di-GMP and other cyclic di-nucleotides are not necessarily recognized by surface receptors (McWhirter et al. 2009). Subsequently cyclic di-GMP and alternative cyclic di-nucleotides have been demonstrated to be efficient noncytotoxic adjuvants when subcutaneously and mucosal administered (Ebensen et al. 2007, Libanova et al. 2010). Cyclic di-GMP has been used as an adjuvant not only with model antigens to stimulate innate and adaptive immune responses, but cyclic di-GMP and/or alternative cyclic di-nucleotides have been demonstrated to protect against subsequent challenge with pathogens such as *Mycobacterium tuberculosis*, influenza virus, HIV, and SARS-CoV-2 (Major et al. 2015, Latanova et al. 2018, Yamamoto et al. 2019, Yan and Chen 2021, Yang et al. 2023, Kwon et al. 2025). Cyclic di-GMP is also a potent adjuvant for cancer vaccines (Wu et al. 2018).

The molecular basis of the broad immunostimulatory action of cyclic di-GMP (and other cyclic di-nucleotides) became, however, only obvious when it was demonstrated that the central immune activator STING effectively recognizes cyclic di-GMP, although not being the canonical ligand (Burdette et al. 2011). Subsequently, cyclic GMP–AMP synthease cGAS was shown to synthesize the distinct metazoan analog 2′,3′ cyclic GMP–AMP as the primary STING ligand in response to cytosolic DNA derived from viruses, bacteria and intrinsic sources such as the mitochondrium (Fig. 3B). Of note, cGAS is homologous to the microbial CBASS CD-NTase DncV showing the conservation of innate immune defense mechanisms from bacteria/archaea to humans (Morehouse et al. 2020). Although binding of cyclic di-GMP, cyclic di-AMP and cyclic GMP-AMP occurs to the same receptor in the case of the central innate immune activator STING (Ergun et al. 2019), the binding sites, affinity and conformational consequences of binding various cyclic di-nucleotides are different. Very recently the human adenylate cyclase 7 ADCY7 has been shown to also act as a di-adenylate cyclase specifically in the pathway downstream of the CpG activated toll-like receptor Tlr9 activating the NLRP3 inflammasome (a receptor for cyclic di-AMP) in the human system (Liu et al. 2025). Consequently, to no surprise, different cyclic di-nucleotides previously showed distinct immune responses (Libanova et al. 2010, Ebensen et al. 2011, Blaauboer et al. 2015).

## Cyclic di-GMP in cancer treatment

Cyclic di-nucleotides being suitable therapeutic agents against cancer is based on their dual effect on the growth inhibition of cancer cells combined with the ability to trigger substantial innate and adaptive immune responses (Corrales et al. 2015, Demaria et al. 2015, Ahn et al. 2018). To optimize cyclic di-GMP and other cyclic di-nucleotides as therapeutic agents, also to avoid cytotoxicity upon overdosage, various delivery modes for cyclic di-nucleotides have been developed. These include effective cytosolic delivery such as the inclusion of cyclic di-nucleotides in different types of liposomes, nanoparticles, modified polysaccharide based microparticles, peptide-based hydrogels, divalent metal ions (Mn^2+^), and amyloids (Miyabe et al. 2014, Hanson et al. 2015, Nakamura et al. 2015, An et al. 2018, Chen et al. 2018, Leach et al. 2018, Sun et al. 2021, Yu et al. 2022). In addition, nuclease-resistant nonhydrolysable analogs of cyclic di-nucleotides ensure a longer lasting effect of the delivered cyclic di-nucleotides (Eckstein 2014, Meric-Bernstam et al. 2022). Alternatively, agonists with alternative chemical structure of central immune activators such as STING can be effective *in vivo* (Pan et al. 2020). Potentials and challenges in developing cyclic di-nucleotides as therapeutics including the biocatalysis of modified nucleotides have been recently reviewed (Wang 2021, Van et al. 2023). Microbes, in particular bacteria and viruses can be effective antitumor agents. Indeed, in the nineteenth century severe bacterial infections have been first observed to retard tumor growth (Coley 1991). Consequently, an alternative to the direct application of cyclic di-GMP, its analogs and semisynthetic analogs is the delivery of cyclic di-nucleotides by microbes that have been shown to selectively and effectively not only colonize tumors, but contribute to their retardation. Indeed, delivery of *Mycobacterium bovis* in the form of the BCG tuberculosis vaccine strain is today still the most effective treatment against bladder cancer (Lobo et al. 2021). Multiple therapeutic effects of tumor-colonizing microbes such as biofilm formation for persistent colonisation, growth retardation of tumor cells, and immunostimulation might combine for enhanced effectiveness in tumor retardation (Crull et al. 2011, Um et al. 2023). Of note, orally administered BCG overexpressing a cyclic di-nucleotide synthase also protects more effectively against *M. tuberculosis* challenge (Dey et al. 2020, Singh et al. 2021).

## Perspective

Optimization of biotechnological processes and therapeutic treatment strategies is essential. In this context, the application of microbes in efficient, cost effective and resource and energy saving applications and in targeting therapeutics minimizing side effects has been and is a research priority. The application of cyclic di-GMP and other cyclic di- and oligo-nucleotides as targets and therapeutic agents and the exploitation of cyclic di-GMP and its signaling pathway components in alternative biotechnological and clinical applications has still promising farther future potentials from wastewater to cancer treatment.

## Supplementary Material

qvaf016_Supplemental_File

## Data Availability

There are no new data associated with this article.
