# Supplementary material for: Impact and applications of cyclic di-GMP second messenger signaling in biotechnology and medicine
Source: Sustain Microbiol. 2025 Jul 24;2(3):qvaf016. doi: 10.1093/sumbio/qvaf016 (PMC13375341; doi:10.1093/sumbio/qvaf016)
Supplement: Supplementary file 1 [file qvaf016_Supplemental_File.pdf]

Supplementary Data

Impact and applications of cyclic di-GMP second messenger signaling in biotechnology and medicine

Ute Römling

Department of Microbiology, Tumor and Cell Biology, Karolinska Institutet, SE-171 77 Stockholm, Sweden

Running head: Cyclic di-nucleotides in applications

Mailing address: Ute Römling, PhD, Department of Microbiology, Tumor and Cell Biology, Biomedicum C8/9, Karolinska Institutet, Solnavägen 9, SE-171 77 Stockholm, Sweden
E-mail: ute.romling@ki.se

Glossary

Biotechnology

Bioflotation: uses physical, metabolic and biochemical properties of microbes to separate, enzymatically process and detoxify solid compounds relevant for the mining and mineral processing industry.

Biotransformation: use of microbes to carry out chemical reactions to obtain bioactive compounds.

Flocculation: (Bio)flocculation is the process of loose association of multiple microorganisms with environmental organic matter in liquid leading to floating aggregates.

Flocculants: also defined as clarifying agents are solid larger particles that aid the aggregation of dispersed smaller solid compounds to associate for efficient removel from liquids

Granules: are tightly interacting self-organized microbial consortia with distinct metabolic properties derived from sludge.

PDB: Protein Data Bank collects experimentally derived 3D-structures of proteins.

Microbiology

Anammox: The ANaerobic AMMonium OXidation reaction $NH_4^+ + NO_2^- > N_2 + 2\ H_2O$ carried out inside the membrane-enclosed anammoxosome by member genera of the phylum Planctomycetetota is exploited as an energy efficient process for denitrification in waste water treatment.

BCG: Bacillus Calmette–Guérin is an attenuated *Mycobacterium bovis* strain that used as a vaccine against *Mycobacterium tuberculosis*.

CBASS: The DncV-like Cyclic Dinucleotide NucleotidylTransferase CD-NTase of the Cyclic Nucleotide-Based Antiphage Signaling Systems is the microbial ancestor of the metazoan cyclic GMP-AMP synthase (cGAS). cGAS together with the effector STING compose the central innate immune signaling system against intruding viruses sensing and responding to nucleic acids.

CRISPR-Cas: Clustered Regularly Interspaced Short Palindromic Repeats-CRISPR associated protein is an adaptive immune system of bacteria and archaea against phages and plasmids.

Cyclic di-AMP: 3',3' cyclic deadenylate is a ubiquitous second messenger molecule identified in bacteria, archaea and humans.

Cyclic di-GMP: 3',3' cyclic di-guanylate is a ubiquitous bacterial second messenger regulating the life style switch between sessility and motility

CZB: Chemoreceptor Zink-Binding signal binding domain.

DAC: Diadenylate cyclase domain

DSF: Diffusible Signaling Factors are cis-2-unsaturated fatty acids derived quorum sensing molecules.

EPS: Extracellular Polymeric Substances of diverse chemical structure. Included are exopolysaccharides, proteins (pili (fimbriae), amyloids and enzymes), extracellular DNA and lipids.

GGDEF/EAL/HD-GYP domains: protein domains involved in turnover of the second messenger cyclic di-GMP named after conserved motifs. GGDEF domains display diguanylate cyclase activity, while EAL

and HD-GYP domains are mainly cyclic di-GMP specific phosphodiesterases with pGpG and GMP as products.

HSAF: Heat Stable Antifungal Factor dihydromaltophilin produced by *Lysobacter enzymogenes.*

PilZ: A conserved and abundant cyclic di-GMP binding domain, first one identified.

Immunology

CD-NTase: Cyclic Dinucleotide-NucleotidylTransferase; cGAS/DncV-like enzymes

CRAMP: Cathelicidin-Related AntiMicrobial Peptide is a murine anti-microbial, anti-biofilm and immunostimulatory peptide with LL-37 being the human equivalent.

cGAS: cyclic GMP-AMP synthase

HIV: Human Immunodeficiency Virus

MAMP: Microbial-Associated Molecular Pattern are conserved microbial immunostimulatory molecules such as the diverse cyclic di-nucleotides.

NLRP3: NLRP3 is the sensor (receptor) of the cytosolic NLRP3 inflammasome complex that activates the proteinase caspase 1 in response to activating signals such as potassium and cyclic di-AMP.

NO: nitric oxide

PAMP: Pathogen-Associated Molecular Pattern are conserved microbial immunostimulatory molecules partially overlapping with MAMPs

SMODS: Second Messenger Oligonucleotide or Dinucleotide Synthetase

SARS-CoV-2: Severe Acute Respiratory Syndrome Corona Virus 2

STING: Stimulator of INterferon Genes, a membrane-anchored cyclic di-nucleotide receptor of the endoplasmatic reticulum, is a central activator of the cyclic AMP-GMP mediated human innate immune response.

TLR: Toll-like receptors are membrane-spanning innate immune components sensing microbial associated molecular pattern molecules such as unmethylated CpG (TLR9) and flagellin the major subunit of bacterial flagella (TLR5). In humans TLR1-TLR10 have been recognized.
